# Associated factors with voriconazole plasma concentration: a systematic review and meta-analysis

**DOI:** 10.3389/fphar.2024.1368274

**Published:** 2024-08-23

**Authors:** Xiaoqi Li, Qiaozhi Hu, Ting Xu

**Affiliations:** Department of Pharmacy, West China Hospital, Sichuan University, Chengdu, Sichuan, China

**Keywords:** voriconazole, drug monitoring, voriconazole plasma concentration, factors, meta-analysis

## Abstract

**Background:** Voriconazole plasma concentration exhibits significant variability and maintaining it within the therapeutic range is the key to enhancing its efficacy. We conducted a systematic review and meta-analysis to estimate the prevalence of patients achieving the therapeutic range of plasma voriconazole concentration and identify associated factors.

**Methods:** Eligible studies were identified through the PubMed, Embase, Cochrane Library, and Web of Science databases from their inception until 18 November 2023. We conducted a meta-analysis using a random-effects model to determine the prevalence of patients who reached the therapeutic plasma voriconazole concentration range. Factors associated with plasma voriconazole concentration were summarized from the included studies.

**Results:** Of the 60 eligible studies, 52 reported the prevalence of patients reaching the therapeutic range, while 20 performed multiple linear regression analyses. The pooled prevalence who achieved the therapeutic range was 56% (95% CI: 50%–63%) in studies without dose adjustment patients. The pooled prevalence of adult patients was 61% (95% CI: 56%–65%), and the pooled prevalence of children patients was 55% (95% CI: 50%–60%) The study identified, in the children population, several factors associated with plasma voriconazole concentration, including age (coefficient 0.08, 95% CI: 0.01 to 0.14), albumin (−0.05 95% CI: −0.09 to −0.01), in the adult population, some factors related to voriconazole plasma concentration, including omeprazole (1.37, 95% CI 0.82 to 1.92), pantoprazole (1.11, 95% CI: 0.17–2.04), methylprednisolone (−1.75, 95% CI: −2.21 to −1.30), and dexamethasone (−1.45, 95% CI: −2.07 to −0.83).

**Conclusion:** The analysis revealed that only approximately half of the patients reached the plasma voriconazole concentration therapeutic range without dose adjustments and the pooled prevalence of adult patients reaching the therapeutic range is higher than that of children. Therapeutic drug monitoring is crucial in the administration of voriconazole, especially in the children population. Particular attention may be paid to age, albumin levels in children, and the use of omeprazole, pantoprazole, dexamethasone and methylprednisolone in adults.

**Systematic Review Registration:** https://www.crd.york.ac.uk/prospero/display_record.php?ID=CRD42023483728.

## 1 Introduction

The issue of invasive fungal infections is significant in contemporary medicine, notably impacting human health, especially among patients with compromised immune systems, resulting in increased morbidity and mortality (Hope et al., 2013). Voriconazole, a second-generation triazole, demonstrates broad-spectrum antifungal activity against *Candida*, C. neoformans, Aspergillus, various dimorphic fungi, and other medically significant fungi (Sabatelli et al., 2006). As an azole antifungal agent, it is prescribed for treating and preventing invasive fungal infections, particularly invasive aspergillosis (Tissot et al., 2017; Walsh et al., 2008; Patterson et al., 2016).

Voriconazole demonstrates linear pharmacokinetics in children, whereas its pharmacokinetics are nonlinear in adults (Chen X. et al., 2022; Theuretzbacher et al., 2006). As the dosage increases, the clearance of the drug does not necessarily increase linearly, potentially being affected by saturation effects or other enzymatic activities (Ludden, 1991). This non-linearity renders the drug more complex and increases the challenge of adjusting dosages and predicting drug concentrations in clinical settings. In addition to its non-linear pharmacokinetics, drug interactions, CYP2C19 genotype, and liver function are potential influences on plasma levels of voriconazole. A correlation between voriconazole exposure and response has been confirmed (Dolton and Mclachlan, 2014). Low voriconazole concentration may elevate the risk of treatment failure, while higher concentrations correlate with heightened toxicity (Elewa et al., 2015). Consequently, maintaining an ideal voriconazole plasma concentration is important but difficult in both children and adults.

A randomized controlled trial indicated significantly better outcomes (complete or partial response) in patients receiving therapeutic drug monitoring (TDM) compared to those not receiving TDM (Park et al., 2012). Therefore, performing TDM on voriconazole is helpful in maintaining the ideal plasma concentration of voriconazole. For TDM of voriconazole, trough concentration is commonly measured (Ashbee et al., 2014). The recommended therapeutic range of voriconazole plasma concentration varies in different regions. A meta-analysis suggested that maintaining the trough concentration of voriconazole between 0.5 and 3.0 mg/L optimizes clinical efficacy while minimizing hepatotoxicity (Jin et al., 2016). Substantial studies indicated that voriconazole trough concentrations maintained above 1.0 mg/L and below 4.0 mg/L are effective and safe (Hamada et al., 2012; Troke et al., 2011; Jeans et al., 2012; Ashbee et al., 2014). These studies (Hamada et al., 2012; Troke et al., 2011; Jeans et al., 2012; Ashbee et al., 2014; Park et al., 2012; Jin et al., 2016), highlight that maintaining specific therapeutic ranges of plasma voriconazole concentration increases the likelihood of therapeutic success. Early TDM to achieve specific range contributes significantly to treatment success (Resztak et al., 2021).

Multiple factors have been confirmed to be associated with interindividual variability in voriconazole concentrations (e.g., CYP2C19 genotype), and several cross-sectional studies have investigated the prevalence of patients achieving therapeutic range and the factors associated with voriconazole plasma concentration. However, there are no evidence-based medical studies summarizing the prevalence of patients reaching the specific therapeutic range of plasma concentration the factors affecting the plasma concentration of voriconazole. Therefore, this systematic review and meta-analysis aggregated data to estimate the prevalence of patients achieving the therapeutic range of plasma voriconazole concentration. Additionally, it analyzed various factors influencing plasma voriconazole levels. The findings could assist clinicians in making more informed treatment decisions. With further research and the accumulation of medical evidence, clinicians can optimize voriconazole use to improve patient outcomes and survival rates.

## 2 Methods

This study was conducted according to the Meta-analysis of Observational Studies in Epidemiology (MOOSE) guidelines (Stroup et al., 2000) and followed the Preferred Reporting Items for Systematics Reviews and Meta-Analyses guidelines (Moher et al., 2015). The protocol of this review was registered in PROSPERO (CRD42023483728).

### 2.1 Search strategy

A preliminary scoping search was conducted in PubMed, the Cochrane Library, Web of Science, and Embase from their inception until 18 November 2023. The search strategy employed a combination of Medical Subject Headings (MeSH) terms and free-text terms. Detailed search strategies are shown in Supplementary Table S1.

### 2.2 Selection criteria

Studies were eligible for inclusion if they: 1) involved patients undergoing voriconazole treatment; 2) reported the number of patients achieving therapeutic plasma concentrations of voriconazole or provided coefficients and standard errors from multiple linear regression analyses; 3) were published in English; and 4) utilized cross-sectional or cohort study designs. Exclusion criteria were 1) conference abstracts, reviews, letters, or commentaries; and 2) studies where data extraction was infeasible or if the independent variable in the multiple linear regression analysis was not clearly defined.

### 2.3 Study selection

Two reviewers (XQ Li and QZ Hu) independently screened the titles and abstracts of the preliminarily included studies, adhering to the predefined inclusion and exclusion criteria. In instances where a study’s eligibility was unclear from the abstract alone, a comprehensive assessment of the full text was conducted. Any discrepancies during the screening process were resolved by consulting a third senior investigator (T Xu).

### 2.4 Data extraction

Data extraction from the included literature was independently conducted by two reviewers (XQ Li and QZ Hu). The following information was collected: author(s), publication year, country of study, study type, patient age and sex demographics, type of treatment settings, method of voriconazole measurement, duration of treatment, number of patients enrolled, number of blood samples analyzed, number of patients achieving the therapeutic range of plasma voriconazole concentration, the execution of multiple linear regression analyses, and, where applicable, coefficients and standard errors derived from these regressions.

### 2.5 Quality assessment

Two reviewers (XQ Li and QZ Hu) assessed the quality of the included studies using the Agency for Healthcare Research and Quality (AHRQ) criteria, which comprise 11 items. Each item was rated as “yes,” “no,” or “unclear.” A “yes” response earned 1 point, while a “no” or “unclear” received no points. Based on their total scores, the studies were categorized into three quality levels: 0–3 indicated low quality, 4–7 moderate quality, and 8–11 high quality.

### 2.6 Statistical analysis

A meta-analysis was conducted to evaluate the prevalence of patients who achieved the therapeutic range of plasma voriconazole concentration, using STATA version 15. The pooled prevalence was reported proportionally, with 95% confidence intervals (CIs). Heterogeneity between studies was assessed using the χ2 test and the I^2^ statistic, with an I^2^ value >50% suggesting significant heterogeneity. Accordingly, the Der Simonian-Laird random-effects model was applied to analyze such cases. A sensitivity analysis was performed to ensure the robustness of the meta-analysis findings. The subgroup analysis was performed based on the study cohort (adults vs. children) and the method of dose adjustments. The potential for publication bias was evaluated using Egger’s test, Begg’s test, and the trim-and-fill method. Additionally, for instances where two or more studies with children or adults population reported coefficients and standard errors (SEs) from multiple linear regression analyses, we summarized these coefficients and SEs using a random-effects model, following the approach outlined by Rolden et al. (2014).

## 3 Result

### 3.1 Study selection

**FIGURE 1:**
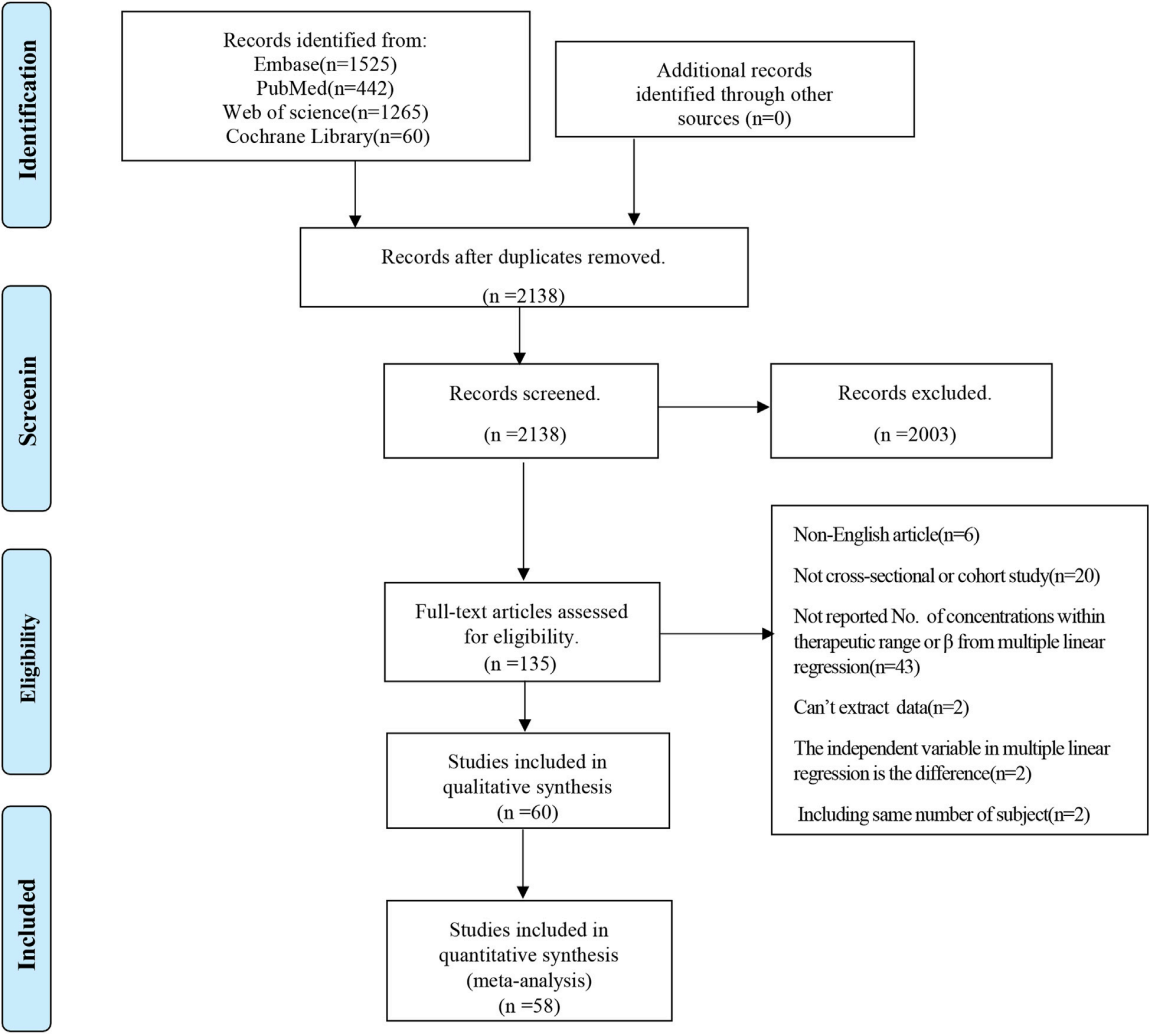
PRISMA flow diagram.

A comprehensive search across four databases yielded 3,292 literature references. Following the exclusion of 1,154 duplicates, 2,138 studies underwent title and abstract screening. Ultimately, 60 studies were selected for qualitative synthesis based on a full-text review (Aiuchi et al., 2022; Allegra et al., 2018; Bartelink et al., 2013; Benedict et al., 2023; Blanco-Dorado et al., 2019; Boast et al., 2016; Cabral-Galeano et al., 2015; Chaudhri et al., 2020; Chen C. Y. et al., 2022; Chen J. et al., 2022; Chen T. T. et al., 2022; Chen X. et al., 2022; Cheng et al., 2020; Choi et al., 2013; Chuwongwattana et al., 2016; Cojutti et al., 2016; Dolton et al., 2012; Dorado et al., 2020; Dote et al., 2016; Duehlmeyer et al., 2021; Ebrahimpour et al., 2017; Fan et al., 2022; Hashemizadeh et al., 2017; Hoenigl et al., 2013; Hu et al., 2018; Hu et al., 2023; Huang et al., 2023; Huang et al., 2020; Jia et al., 2021; Kang et al., 2015; Kim et al., 2014; Lempers et al., 2019; Li et al., 2020; Li et al., 2023; Liu et al., 2017; Mafuru et al., 2021; Miao et al., 2019; Myrianthefs et al., 2010; Pieper et al., 2012; Ronda et al., 2023; Ruiz et al., 2019; Saini et al., 2014; Shao et al., 2017; Shen et al., 2022; Soler-Palacin et al., 2012; Takahashi et al., 2020; Tian et al., 2021; Troke et al., 2011; Valle-T-Figueras et al., 2021; Wei et al., 2019; Yan et al., 2018; Yang et al., 2023; Ye et al., 2022; Yi et al., 2017; Zeng et al., 2020; Zhang et al., 2023; Zhao et al., 2021a; Zhao et al., 2021b; Zhao Y. C. et al., 2021; Zhou et al., 2020). Of the 60 articles, 58 studies were included in the quantitative synthesis. Figure 1 shows the specific screening process of the literature.

### 3.2 Study characteristics

All included studies were cross-sectional studies. The characteristics of the included studies are detailed in Table 1. Of these, 20 studies performed multiple linear regression and reported coefficients (Allegra et al., 2018; Chen T. T. et al., 2022; Chen W. Q. et al., 2022; Cojutti et al., 2016; Dolton et al., 2012; Dorado et al., 2020; Dote et al., 2016; Fan et al., 2022; Hashemizadeh et al., 2017; Hu et al., 2018; Hu et al., 2023; Mafuru et al., 2021; Ronda et al., 2023; Takahashi et al., 2020; Tian et al., 2021; Ye et al., 2022; Zeng et al., 2020; Zhao et al., 2021a; Zhao et al., 2021b; Zhao Y. C. et al., 2021). In contrast, 53 studies provided data on the number of patients achieving the therapeutic range of plasma voriconazole concentration (Aiuchi et al., 2022; Bartelink et al., 2013; Benedict et al., 2023; Boast et al., 2016; Cabral-Galeano et al., 2015; Chaudhri et al., 2020; Chen C. Y. et al., 2022; Chen J. et al., 2022; Chen T. T. et al., 2022; Chen X. et al., 2022; Cheng et al., 2020; Choi et al., 2013; Chuwongwattana et al., 2016; Dorado et al., 2020; Duehlmeyer et al., 2021; Ebrahimpour et al., 2017; Hoenigl et al., 2013; Hu et al., 2018; Hu et al., 2023; Huang et al., 2023; Huang et al., 2020; Jia et al., 2021; Kang et al., 2015; Kim et al., 2014; Lempers et al., 2019; Li et al., 2020; Li et al., 2023; Miao et al., 2019; Myrianthefs et al., 2010; Pieper et al., 2012; Ronda et al., 2023; Ruiz et al., 2019; Saini et al., 2014; Shao et al., 2017; Shen et al., 2022; Soler-Palacin et al., 2012; Takahashi et al., 2020; Tian et al., 2021; Troke et al., 2011; Valle-T-Figueras et al., 2021; Wei et al., 2019; Yan et al., 2018; Yang et al., 2023; Ye et al., 2022; Yi et al., 2017; Zeng et al., 2020; Zhang et al., 2023; Zhao et al., 2021b; Zhao Y. C. et al., 2021; Zhou et al., 2020; Blanco-Dorado et al., 2020).

**TABLE 1:** Characteristics of included studies.

Study	Country	Type	Age (mean or median)	Male (%)	Type of treatment setting	Method	Indications	Duration (days) (mean or median)	No. of patients (samples)	Dose adjustments	No. of samples within therapeutic range (%)	Range (mg/L)	Multiple linear regression
Aiuchi et al. (2022)	Japan	Cross-sectional	67.2 ± 14.1	—	—	UPLC-MS/MS	Prophylaxis treatment	—	36 (56)	No	35 (62.5)	1.0–5.0	No
Allergra et al. (2018)	Italy	Cross-sectional	10 (7–14)	62.9	—	HLPC-MS/MS	Prophylaxis treatment	—	233 (233)	—	—	—	Yes
Bartelink et al. (2013)	Nether lands	Cross-sectional	7.3 (0.3–20)	59	Pediatric HSCT	HPLC	Prophylaxis treatment	39 (6–415)	61 (61)	Yes	49 (80.4)	1.0–5.0	No
Benedict et al. (2023)	America	Cross-sectional	58.0 (42–68)	—	—	—	Treatment	—	296 (296)	Yes	186 (62.9)	1.0–5.5	No
Blanco-Dorado et al. (2019)	Spain	Cross-sectional	68 (19–93)	55.1	—	HPLC	Prophylaxis treatment	26 (4–185)	78 (78)	No	34 (44.8)	1.0–5.5	No
Boast et al. (2016)	Austria	Cross-sectional	10.5	—	—	HPLC	Prophylaxis treatment	72.5 (4–567)	55 (120)	Yes	53 (44.2)	1.0–5.0	No
Cabral-Galeano et al. (2015)	Spain	Cross-sectional	55 (35.7–60.7)	59.6	—	HPLC	Prophylaxis treatment	56 (21–98)	52 (52)	Yes	36 (69.2)	1.0–5.5	No
Chaudhri et al. (2020)	Australia	Cross-sectional	57 (21–89)	55.6	—	HPLC	Prophylaxis treatment	9 (1–64)	90 (101)	Yes	58 (57)	1.0–5.0	No
Chen C. Y. et al. (2022)	China	Cross-sectional	59 (47–68)	65.5	—	HPLC	Prophylaxis treatment	15 (8–16.5)	325 (325)	No	217 (66.8)	1.0–5.0	No
Chen J. et al. (2022)	China	Cross-sectional	6.0 (3.0–9.0)	59.3	Pediatric HSCT	HLPC-MS/MS	NA	59 (23–149)	91 (682)	Yes	422 (61.9)	1.0–5.0	No
Chen T. T. et al. (2022)	China	Cross-sectional	39.71 ± 14.14	85.7	AIDS	HPLC	Treatment	—	28 (46)	Yes	16 (57.1)	1.0–5.5	Yes
Chen X. et al. (2022)	China	Cross-sectional	6 (1–15)	60.6	Pediatrics	2D-HPLC-MS/MS	Prophylaxis treatment	—	94 (253)	No	40 (42.6)	2.0–5.0	Yes
Cheng et al. (2020)	China	Cross-sectional	55.4	64.5	—	LC-MS/MS	Prophylaxis treatment	—	166 (317)	Yes	213 (67.2)	1.0–5.0	No
Choi et al. (2013)	Korea	Cross-sectional	12.2 (1.2–18.9)	66.7	Oncology	HLPC-MS/MS	Prophylaxis treatment	60 (10–305)	27 (193)	Yes	123 (63.7)	1.0–6.0	No
Chuwongwattana et al. (2016)	Thailand	Cross-sectional	57 (18–88)	53	—	LC-MS/MS	Treatment	—	115 (285)	NA	151 (52.98)	1.0–4.0	No
Cojutti et al. (2016)	Italy	Cross-sectional	54 (41–62)	61.4	Hematology	HLPC-MS/MS	Prophylaxis treatment	36 (18–81)	83 (199)	—	—	1.0–5.5	Yes
Dolton et al. (2012)	Australia	Cross-sectional	54 (18–88)	57.7	—	HPLC	Prophylaxis treatment	—	201 (783)	—	—	—	Yes
Dorado et al. (2020)	Spain	Cross-sectional	68 (19–93)	55.1	—	HPLC	Prophylaxis treatment	—	78 (78)	No	34 (44.8)	1.0–5.5	Yes
Dote et al. (2016)	Japan	Cross-sectional	70.8 ± 11.0	79.4	Hematology Pulmonology Rheumatology Other	HPLC	Prophylaxis treatment	—	63 (77)	—	—	—	Yes
Duehlmeyer et al. (2021)	America	Cross-sectional	<20	—	—	—	Treatment	—	39 (77)	Yes	39 (50.7)	2–5.5	No
Ebrahimpour et al. (2017)	Iran	Cross-sectional	34.6 ± 11.2	51.4	Hematology-oncology and stem cell research center	HPLC	NA	—	37 (37)	No	27 (73.0)	1.0–5.5	No
Fan et al. (2022)	China	Cross-sectional	6.5 ± 3.7	55.9	Hematology	UPLC-MS/MS	Prophylaxis treatment	—	68 (68)	—	—	—	Yes
Hashemizadeh et al. (2017)	Iran	Cross-sectional	36 ± 13.71	42.3	Liver transplant center	HPLC	Treatment	39 (21–50)	104 (832)	—	—	1.0–5.5	Yes
Hoenigl et al. (2013)	Austria	Cross-sectional	55.94	59	ICU Hematological malignancy	HPLC	Prophylaxis treatment	—	61 (221)	Yes	79 (36)	1.5–5.5	No
Hu et al. (2018)	China	Cross-sectional	9 (3–14)	51.7	Hematology	HPLC	Prophylaxis treatment	23.5 (4–159)	42 (138)	Yes	28 (66.7)	1.0–5.5	Yes
Hu et al. (2023)	China	Cross-sectional	13 (2–14)	61.1	Pediatric hematology	HPLC	Prophylaxis treatment	15 (3–148)	131 (250)	Yes	129 (51.6)	1.0–5.5	Yes
Huang et al. (2023)	China	Cross-sectional	56 ± 14.91	56.6	Hematology	HPLC	Prophylaxis treatment	—	136 (136)	Yes	90 (66.2)	1.0–5.0	No
Huang et al. (2020)	China	Cross-sectional	Control 39.0 (4–68) LAN 39.0 (4–68) OME 44.0 (9–78) PAN 39.5 (3–84)	57.7	Hematology	2D-HPLC	NA	—	194 (194)	Yes	152 (78.4)	1.0–5.5	No
Jia et al. (2021)	China	Cross-sectional	51.47 ± 17.55	58	—	2D-HPLC	NA	—	231 (918)	Yes	714 (77.8)	0.5–5.5	No
Kang et al. (2015)	Korea	Cross-sectional	8.7 ± 6.3	58.1	—	HLPC-MS/MS	Prophylaxis treatment	—	31 (271)	Yes	180 (66.7)	1.0–5.5	No
Kim et al. (2014)	Korea	Cross-sectional	54 (23–81)	67.2	Oncology	—	Prophylaxis treatment	—	64 (354)	Yes	48 (75)	1.0–5.5	No
Lempers et al. (2019)	Nether lands	Cross-sectional	7.0 (1.2–18.5)	38.1	Pediatric hematology-oncology	HPLC-MS/MS	Prophylaxis treatment	118 (17–866)	21 (485)	Yes	282 (58.1)	1.0–6.0	No
Li et al. (2020)	China	Cross-sectional	62 (47.5–76.5)	74.4	ICU	HPLC	Prophylaxis (19.2) treatment (80.8)	14	125 (125)	No	67 (53.6)	1.5–4.0	No
Li et al. (2023)	China	Cross-sectional	alb < 35 g/L:61 alb ≥ 35 g/L:50	59.2	—	LC-MS/MS	Prophylaxis treatment	—	120 (275)	Yes	174 (71.9)	1.0–5.5	No
Liu et al. (2017)	China	Cross-sectional	2.1 (0.1–11.1)	75.2	Pediatrics	LC-MS/MS	Prophylaxis treatment	—	107 (126)	No	51 (47.7)	1.0–5.5	No
Mafuru et al. (2021)	China	Cross-sectional	36 (25–51)	72.8	Hematology	LC-MS/MS	Prophylaxis treatment	—	114 (250)	—	—	—	Yes
Miao et al. (2019)	China	Cross-sectional	49.5 (39.5–62.3)	64.2	—	UPLC-MS/MS	NA	—	106 (152)	Yes	107 (71.7)	1.5–5.5	No
Myrianthefs et al. (2010)	Greece	Cross-sectional	62.3 ± 22	80.0	ICU	HPLC	Prophylaxis treatment	—	18 (18)	No	6 (33.3)	1.0–5.5	No
Pieper et al. (2012)	Germany	Cross-sectional	10.2	56.8	Pediatrics	HPLC	Prophylaxis treatment	40 (6–1,002)	74 (251)	NA	86 (34.6)	1.0–5.0	No
Ronda et al. (2023)	Spain	Cross-sectional	58 ± 10	62.5	ICU	HPLC	Prophylaxis treatment	16.5	24 (53)	Yes	26 (49.1)	1.0–5.0	Yes
Ruiz et al. (2019)	Spain	Cross-sectional	55.3 (12.6)	54.5	ICU	EIA	Prophylaxis treatment	—	33 (33)	No	15 (45.5)	1.0–5.5	No
Saini et al. (2014)	Canada	Cross-sectional	58 (19–80)	55.1	Hematology	LC-MS/MS	Treatment	—	69 (69)	No	40 (58.0)	0.5–5.0	No
Shao et al. (2017)	China	Cross-sectional	49.6 ± 18.2	59.3	Hematology	HPLC	Prophylaxis treatment	—	86 (106)	Yes	67 (63.2)	1.0–4.0	No
Shen et al. (2022)	China	Cross-sectional	63 (52–72)	72.9	—	—	Treatment	—	140 (140)	Yes	117 (84.1)	1.5–5.5	No
Soler-Palacin et al. (2012)	Spain	Cross-sectional	10 (1–17)	53.3	Pediatrics	HPLC	Treatment	42 (7–588)	30 (196)	Yes	84 (42.9)	1.0–5.5	No
Takahashi et al. (2020)	America	Cross-sectional	11.7 ± 5.3	65.9	HSCT	LC-MS/MS	Prophylaxis treatment	—	44 (44)	No	16 (36)	1.5–5.0	Yes
Tian et al. (2021)	China	Cross-sectional	10.5 (0.67–18.0)	63.9	Pediatrics	UPLC-MS/MS	Prophylaxis treatment	—	108 (348)	Yes	221 (63.8)	0.5–5.0	Yes
Troke et al. (2011)	New Zealand	Cross-sectional	44 (12–90)	66.2	—	HPLC	Treatment	—	825 (825)	Yes	591 (71.6)	0.5–5.0	No
Valle-T-Figueras et al. (2021)	Spain	Cross-sectional	9 (6–10)	55.6	—	HPLC	Treatment	80.5 (15–117)	27 (229)	Yes	147 (64.2)	1.0–5.5	No
Wei et al. (2019)	China	Cross-sectional	57 (31–67)	59.7	Hematology ICU	HPLC	Prophylaxis treatment	—	67 (119)	NA	70 (59)	1.0–5.5	No
Yan et al. (2018)	China	Cross-sectional	41.7	75.6	—	2D-HPLC	NA	—	349 (349)	No	258 (74.0)	1.0–5.5	No
Yang et al. (2023)	China	Cross-sectional	66 (57–71)	72.3	Hematology Respiratory medicine Emergency/intensive care medicine Oncology Others	2D-HPLC	Treatment	—	83 (83)	No	66 (79.5)	1.0–5.5	No
Ye et al. (2022)	China	Cross-sectional	58.1 ± 17.0	74.5	ICU	UPLC-MS/MS	Treatment	—	132 (132)	Yes	63 (47.7)	2.0–5.5	Yes
Yi et al. (2017)	America	Cross-sectional	50 (33–60)	49.2	—	HPLC	Treatment	—	122 (250)	Yes	134 (54)	1.0–5.5	No
Zeng et al. (2020)	China	Cross-sectional	40 ± 18	60.5	—	HLPC-MS/MS	Prophylaxis treatment	—	170 (510)	Yes	342 (67.1)	1.0–5.5	Yes
Zhang et al. (2023)	China	Cross-sectional	53.2 ± 13.4	78.8	—	HPLC	Treatment	—	66 (66)	No	48 (72.7)	1.0–5.5	No
Zhao et al. (2021a)	China	Cross-sectional	49.4 ± 11.7	90.7	—	2D-HPLC	Prophylaxis treatment	12 (5–45)	43 (144)	—	—	1.0–5.5	Yes
Zhao et al. (2021b)	China	Cross-sectional	52.0 (40.0–64.0)	70.7	—	2D-HPLC	Treatment	—	676 (1,212)	Yes	741 (61.1)	1.0–5.5	Yes
Zhao Y. C. et al. (2021)	China	Cross-sectional	—	52.1	Pediatric	2D-HPLC	Prophylaxis treatment	—	94 (145)	Yes	78 (53.8)	1.0–5.5	Yes
Zhou et al. (2020)	Singapore	Cross-sectional	56 ± 14	67.1	—	2D-HPLC	Prophylaxis treatment	—	70 (70)	No	32 (45.7)	2.0–5.5	No

Abbreviations: UPLC, ultraperformance liquid chromatography; HPLC, high-performance liquid chromatography system; HPLC-MS, high performance liquid chromatography-mass spectrometry; HPLC-MS/MS, high-performance liquid chromatography-tandem mass spectrometry; LC-MS/MS, liquid chromatography tandem-mass spectrometry; UPLC–QTOF/MS, ultra-performance liquid chromatography quadrupole time of flight mass spectrometry; 2D-HPLC, automatic two-dimensional liquid chromatography; EIA, enzyme immunoassay technique; HSCT, hematopoietic stem cell transplantation; ICU, intensive care unit; PAD, photodiode array detection; LAN, lansoprazole; OME, omeprazole; PAN, pantoprazole; Alb, albumin.

The total count of patients and blood samples in these studies was 7,319 and 14,646, respectively. The recommended concentration ranges for voriconazole varied between studies and included 1.0–5.5 mg/L (n = 24), 1.0–5.0 mg/L (n = 10), 0.5–5.0 mg/L (n = 3), 1.5–5.5 mg/L (n = 3), 2.0–5.5 mg/L (n = 3), 1.0–4.0 mg/L (n = 2), and 1.0–6.0 mg/L (n = 2). Additionally, there was one study each for the ranges 1.5–4.0 mg/L, 0.5–5.5 mg/L, and 1.5–5.0 mg/L. Regarding dose adjustments, 33 studies implemented them, 16 did not, and 3 did not specify. Among the 33 studies that adjusted doses, one followed Australian guideline, three adhered to Chinese guidelines, 16 based adjustments on TDM, clinical efficacy, adverse events, and clinician experience, 13 did not specify the adjustment methodology, and only five studies explicitly recorded their dose adjustment plan. Details of dose adjustments are shown in Supplementary Table S2.

### 3.3 Study quality assessment

The 60 studies included in this analysis were all cross-sectional studies. Regarding the quality assessment based on the AHRQ scoring system, each study scored 5 or higher. Specifically, 4 studies were categorized as high quality, while the remaining 56 were considered moderate quality. Of these, 4 studies scored 8, 30 scored 7, 24 scored 6, and only 2 scored 5. Detailed results of the quality assessment for each study are shown in Supplementary Table S3.

### 3.4 Prevalence of patients reaching the therapeutic range of plasma concentration

**FIGURE 2:**
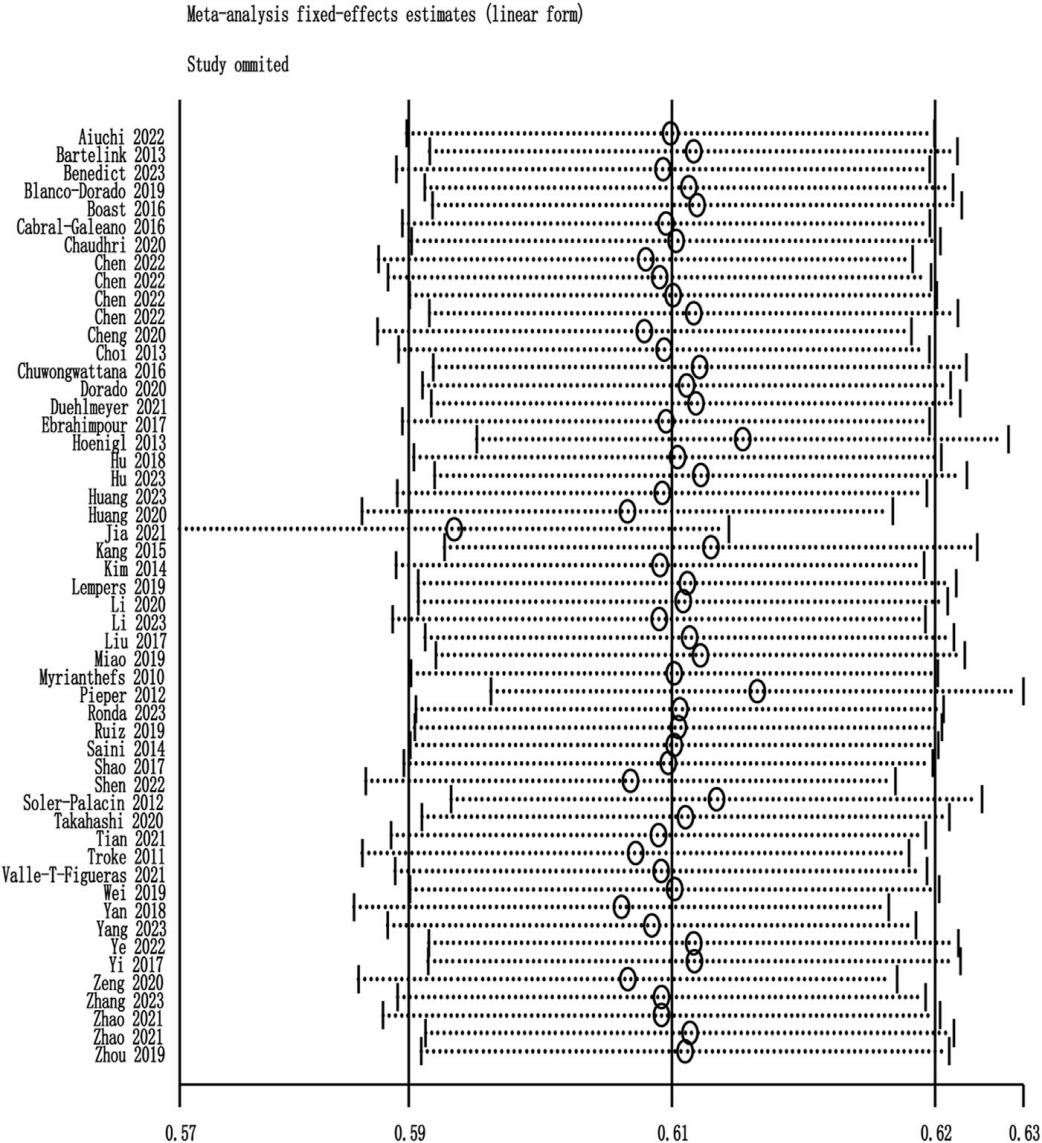
Sensitivity analysis.

**FIGURE 3:**
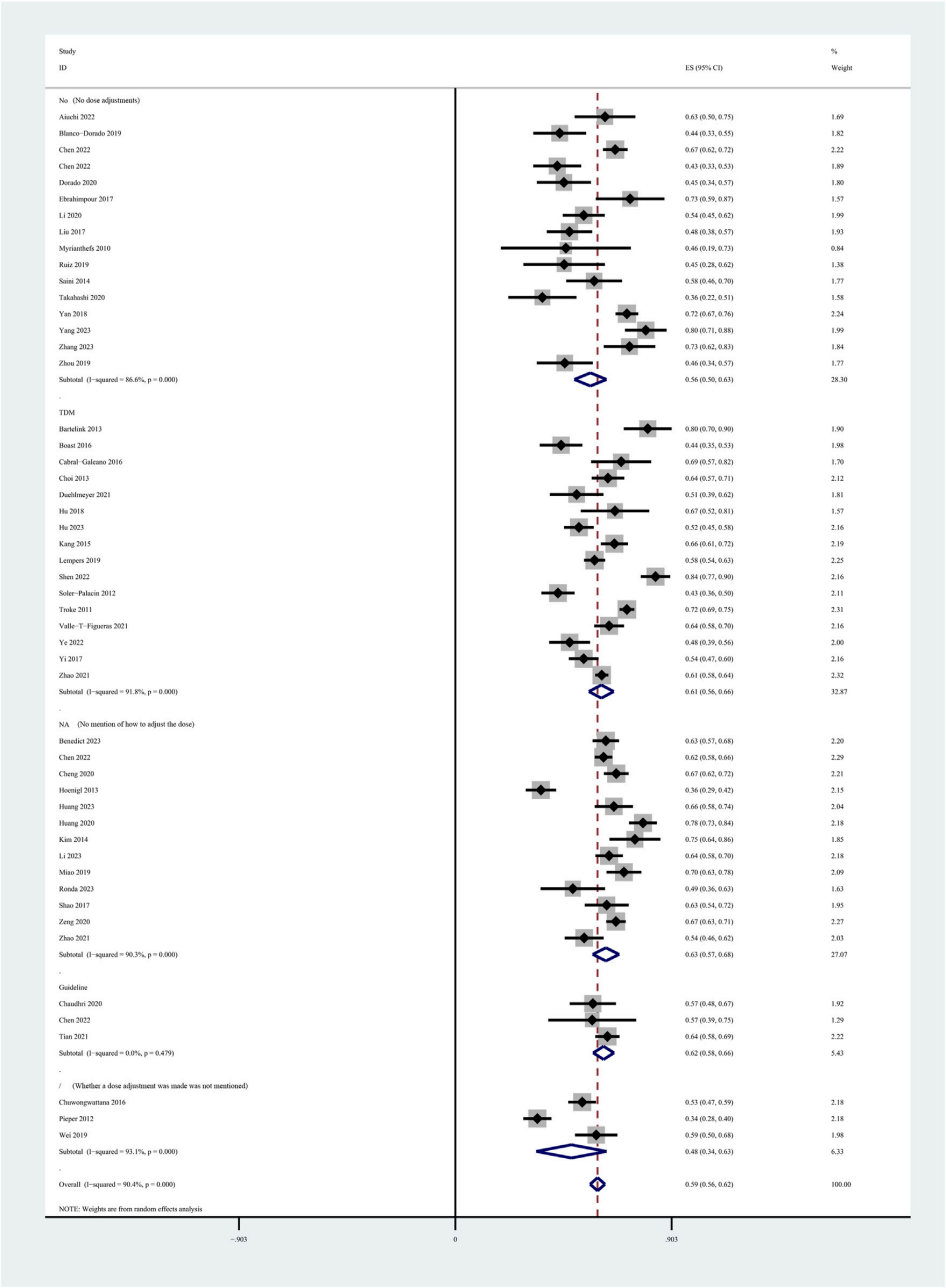
Prevalence of patients reaching the therapeutic range of voriconazole plasma concentration across the method of dose adjustment.

**FIGURE 4:**
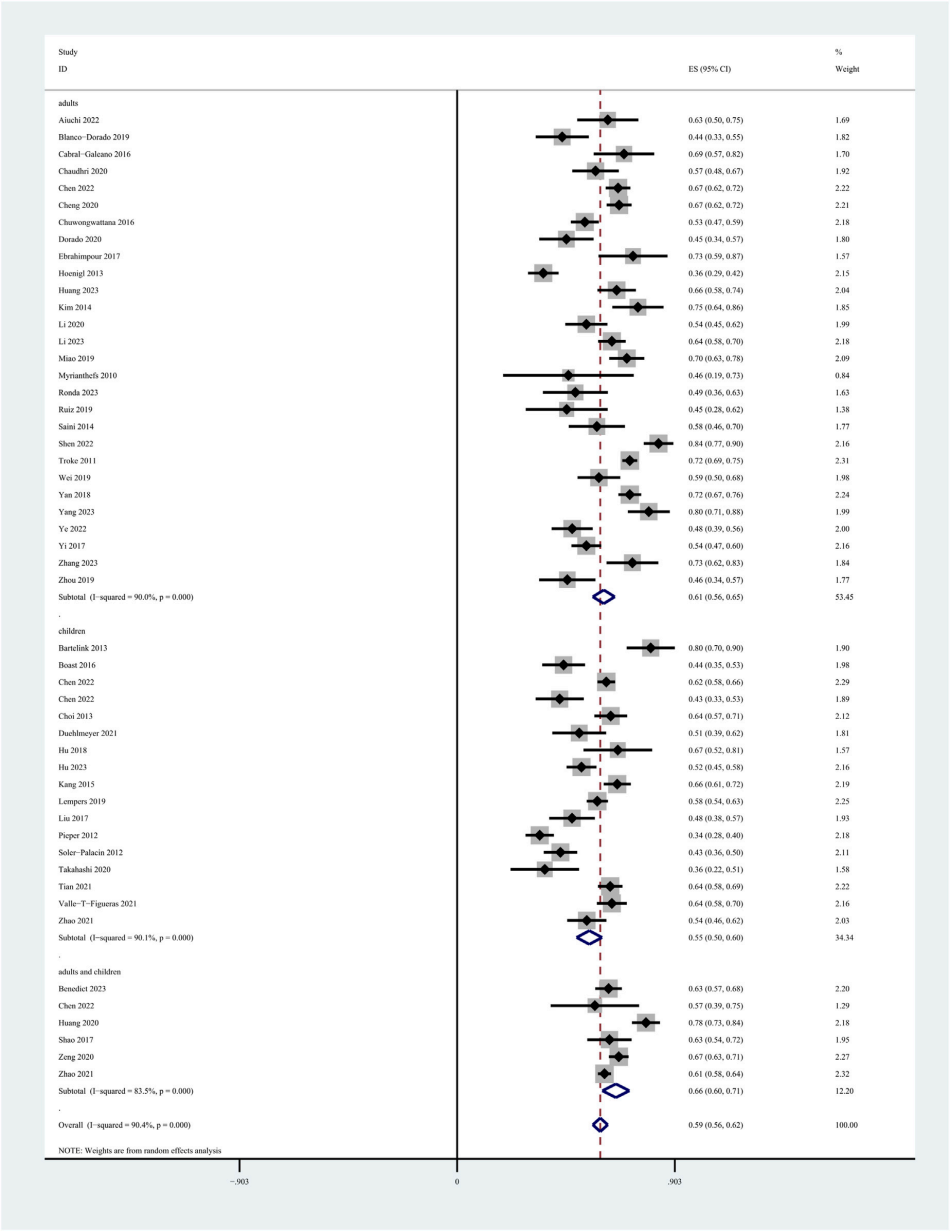
Prevalence of patients reaching the therapeutic range of voriconazole plasma concentration across study cohort.

Among the 60 included studies, 52 reported the prevalence of patients who achieved the plasma voriconazole concentration therapeutic range. The highest and lowest recorded prevalences were 0.84 and 0.33, respectively. A sensitivity analysis led to the exclusion of one study that significantly skewed the results, as shown in Figure 2. After removing this outlier, the pooled prevalence of patients reaching therapeutic voriconazole concentration was 59% (95% CI: 56%–62%). In the subgroup analysis according to the method of dose adjustment, the pooled prevalence was 56% (95% CI: 50%–63%) in studies without dose adjustment and 61% (95% CI: 56%–66%) in studies with dose adjustment according to TDM, and the pooled prevalence of guideline-adjusted dose studies was 62% (95% CI: 58%–66%), as shown in Figure 3. In the subgroup analysis according to study cohort, the pooled prevalence of adult patients was 61% (95% CI: 56%–65%), and the pooled prevalence of children patients was 55% (95% CI: 50%–60%), as shown in Figure 4.

The meta-analysis revealed high heterogeneity in all included studies. To investigate the sources of this heterogeneity, a meta-regression was performed, considering factors such as sex, country, dose adjustments, therapeutic range, type of treatment settings, and the analytical method used for measuring voriconazole. The results identified the therapeutic range, sex, and analytical method as significant contributors to the heterogeneity. Detailed findings from the meta-regression are presented in Supplementary Figure S1.

### 3.5 Associated factors with voriconazole concentration

**FIGURE 5:**
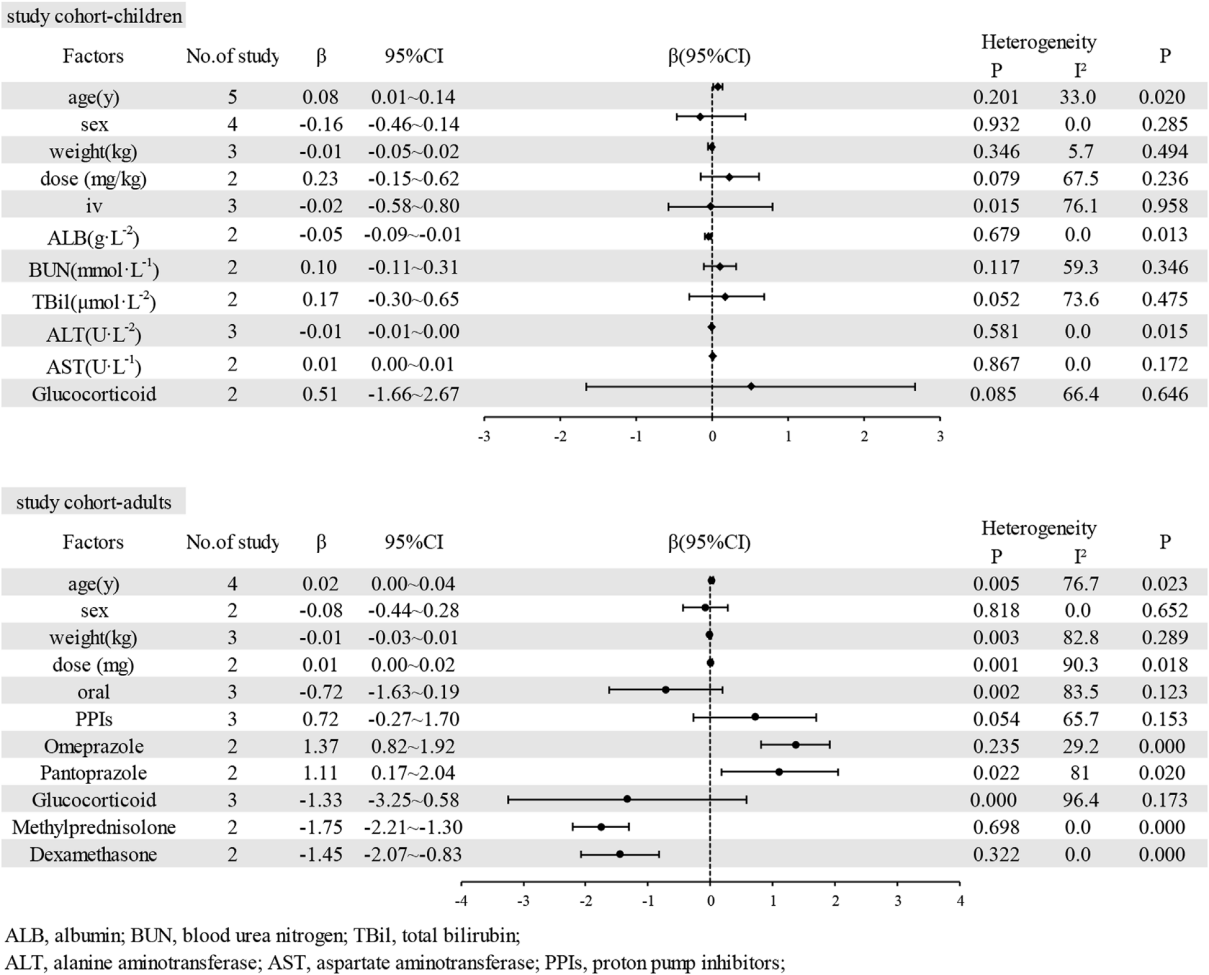
Factors associated with voriconazole plasma concentration.

Of the 60 studies, 20 conducted multiple linear regression and reported coefficients. However, only 17 studies provided coefficients and standard errors derived from regression analyses. Table 2 details the specific factors reported in each study. Due to consistency in units and references between studies, we pooled and analysed respectively the coefficients and standard errors of associated factors about plasma voriconazole concentration in children and adult cohorts. These factors included age, sex, dosage, administration modes (oral and intravenous), albumin (ALB), blood urea nitrogen (BUN), total bilirubin (TBil), alanine aminotransferase (ALT), aspartate aminotransferase (AST), use of proton pump inhibitors (PPIs), omeprazole, pantoprazole, glucocorticoids, methylprednisolone, and dexamethasone. Figure 5 presents details of all the potential confounders identified in these studies.

**TABLE 2:** Factor associated with voriconazole plasma concentration reported by each study.

Variable in multiple linear regression	Risk factors	Studies	No. of patients (concentrations)	Study cohort	R^2^
Sex, ethnicity, voriconazole dose (mg/kg), genetic factors (SLCO1B3, ABCG2, ABCC2 and ABCB1 gene polymorphisms)	Ethnicity, sex, genetic factors (SLCO1B3, ABCG2, ABCC2 and ABCB1 gene polymorphisms)	Allergra et al. (2018)	233	Pediatric patients	—
Drug interaction, ALB (g/L), sex, weight (kg), child-pugh classification	Drug interaction, ALB (g/L), sex, weight (kg), child-pugh classification	Chen T. T. et al. (2022)	28 (46)	AIDS patients	0.406
Age, sex, weight (kg), ALT (U/L) CYP2C19 gene polymorphism, drug administration	Age, CYP2C19 gene polymorphism	Chen W. Q. et al. (2022)	94 (253)	Pediatric patients	—
Omeprazole, pantoprazole, methylprednisolone, dexamethasone, phenobarbital, rifampin, carbamazepine	Omeprazole, pantoprazole, dexamethasone, rifampin, methylprednisolone, carbamazepine phenobarbital	Cojutti et al. (2016)	83 (199)	Adults	
PO, age, weight (kg), daily dose (mg), CYP2C19inducer (phenytoin or rifampin), prednisone, methylprednisolone, dexamethasone, omeprazole, pantoprazole, esomeprazole, rabeprazole	PO, age, weight (kg), daily dose (mg), CYP2C19inducer (phenytoin or rifampin), prednisone, methylprednisolone, dexamethasone, omeprazole, pantoprazole, esomeprazole, rabeprazole	Dolton et al. (2012)	201 (783)	Adults	0.24
Age, sex, weight (kg), PO, PPIs, CYP2C19*2, CYP2C19*17, CYP450 inhibitor combination, CYP450 inductor combination, PPIs, (pantoprazole/omeprazole)	Age	Dorado et al. (2020)	78	Adults	—
ALB (g/dL), CRP (mg/dL), glucocorticoid	ALB (g/dL), CRP (mg/dL), glucocorticoid	Dote et al. (2016)	67 (77)	Patients	—
Age, weight (kg), glucocorticoid, PPIs, CYP2C19*2 allele, CYP2C19*3 allele	CYP2C19*2 allele, CYP2C19*3 allele	*Fan et al. (2022)	68	Pediatric patients	—
Age, weight (kg), PO, sex, PPIs, ^a^CYP2C19 genotype (heterozygous extensive metabolizer, ultrarapid metabolizer, poor metabolizer), glucocorticoids, tacrolimus/cyclosporine, (pantoprazole/omeprazole)	PO, sex, CYP2C19 heterozygous extensive metabolizer (CYP2C19*1/*2, *1/*3, *2/*3, or *2/*17), CYP2C19 ultrarapid metabolizer (CYP2C19*17/*17), CYP2C19 poor metabolizer (CYP2C19*2/*2, *3/*3), glucocorticoids, PPIs (pantoprazole/omeprazole)	Hashemizadeh et al. (2017)	104 (832)	Adult patients undergoing liver transplant	0.932
Age, weight (kg), sex, HSCT, IV, PPIs, glucocorticoids, globulin (U/L), ALB (g/L), TBil (μmol/L), DBil (μmol/L), ALT (U/L), AST (U/L), Scr(μmol/L), BUN (mmol/L)	IV, PPIs	Hu et al. (2018)	42 (138)	Pediatric patients	—
Sex, weight (kg), age, IV, ALB (g/L), ^b^CYP2C19 phenotype (intermediate metabolizer, poor metabolizer), omeprazole, glucocorticoids, TBil (μmol/L), ALT (U/L), AST (U/L), Scr(μmol/L), BUN (mmol/L)	CYP2C19 intermediate metabolizer (CYP2C19*1/*2, *1/*3), CYP2C19 poor metabolizer (CYP2C19*2/*2, *2/*3, or *3/*3), omeprazole, ALB (g/L), ALT (U/L)	Hu et al. (2023)	131 (250)	Pediatric patients	0.138
Age (yr.), csevere aplastic anemia, ^b^CYP2C19 phenotype poor metabolizer, PPIs, ^d^poor metabolizer/PPIs user	Age, severe aplastic anemia, CYP2C19 poor metabolizer (CYP2C19*2/*2, *2/*3, or *3/*3), PPIs (omeprazole/lansoprazole/pantoprazole/esomeprazole), CYP2C19 poor metabolizer/PPIs use combination	Mafuru et al. (2021)	114 (250)	Adult patients with hematologic disorders	—
ALB (g/L), ECMO support, corticosteroids	ALB (g/L), ECMO	Ronda et al. (2023)	24 (53)	Adult patients in ICU	—
Obesity status, age, dose (mg/kg), ^e^CYP2C19 phenotype (normal metabolizer, rapid/ultrarapid metabolizer	Obesity status, age, dose (mg/kg), CYP2C19 normal metabolizer (CYP2C19*1/*1), CYP2C19 rapid (CYP2C19*1/*17)/ultrarapid metabolizer (CYP2C19*17/*17)	Takahashi et al. (2020)	44	Children undergoing HSCT	0.33
Age, PPls non-user, IV, ^e^CYP2C19 phenotype (ultrarapid metabolizer/extensive metabolizers)	Age, CYP2C19 ultrarapid metabolizer (CYP2C19*1/*17, *17/*17)/extensive metabolizers (CYP2C19*1/*1), PPls non-user	Tian et al. (2021)	108 (348)	Children	0.234
Age, APACHE II, SOFA, Scr(μmol/L), dose (mg), PPIs, glucocorticoids, CRRT, ECMO	Sequential organ failure assessment, dose (mg), glucocorticoids, ECMO	*Ye et al. (2022)	132 (132)	Adult patients in ICU	0.322
Loading dose (mg), TBil (μmol/L), PCT (ng/mL), PXRrs3814057	Loading dose (mg), TBil (μmol/L), PCT (ng/mL), PXRrs3814057 polymorphism	Zeng et al. (2020)	170 (510)	Patients with hematological malignancies	0.241
Daily dose (mg), PTA, sex, ^f^CYP2C19 genotyping (*1/*2, *1/*3, *2/*2)	Daily dose (mg), PTA, sex, CYP2C19 genotyping *1/*3, CYP2C19 genotyping*2/*2	Zhao et al. (2021a)	43 (115)	Patient with liver dysfunction	0.348
Age, ALT (U/L), TBil (μmol/L), ALB (g/L), GGT (U/L)	Age, TBil (μmol/L), GGT (U/L)	Zhao et al. (2021b)	676 (1,212)	Patients	0.270
Weight (kg), dose (mg/kg), DBil (μmol/L), urea nitrogen, CYP2C19 phenotype (intermediate metabolizer, poor metabolizer)	Weight (kg), dose (mg/kg), DBil (μmol/L), urea nitrogen, CYP2C19 intermediate metabolizer (CYP2C19*1/*2, *1/*3 or *2/*17), CYP2C19 poor metabolizer (CYP2C19*2/*2, *2/*3, or *3/*3)	*Zhao Y. C. et al. (2021)	94 (145)	Pediatric patients	0.362

^a^ Compared to homozygous extensive metabolizers (CYP2C19*1/*1).

^b^ Compared to normal metabolizer (CYP2C19*1/*1).

^c^ Acute myeloid leukemia.

^d^ Normal metabolizer (CYP2C19*1/*1)/PPIs, non-user.

^e^ Compared to poor metabolizers (CYP2C19*2/*2, *2/*3, or *3/*3)/intermediate metabolizers (CYP2C19*1/*2, *1/*3, or *2/*2).

^f^ CYP2C19 genotyping *1/*1.

*No β95% CI is given.

Abbreviations: ALB, albumin; ALT, alanine aminotransferase; PO, per os; AST, aspartate aminotransferase; CRP, C- reactive protein; PPIs, proton pump inhibitors; TBil, total bilirubin; DBil, direct bilirubin; Scr, serum creatinine; BUN, blood urea nitrogen; IV, intravenous; GGT, gamma glutamyl transferase; SOFA, sequential organ failure assessment; PCT, procalcitonin; PTA, prothrombin time activity.

#### 3.5.1 Age

For the children cohort, among the 21 studies, 6 examined the association between age and voriconazole plasma concentration. However, due to one study did not report the standard error of its coefficient, we pooled the coefficients of only 5 studies. The resulting pooled coefficient was 0.08 (95% CI: 0.01–0.14), indicating a statistically significant association. For the adult cohort, 5 of the 20 studies explored the correlation between age and plasma concentration of voriconazole. However, as one study lacked the report of its coefficient’s standard error, we combined only the coefficients from 4 studies. The pooled coefficient for this analysis was 0.02 (95% CI: 0.00–0.04), but this result was not statistically significant.

#### 3.5.2 Sex

In terms of sex (females), four studies investigated its relationship with voriconazole plasma concentration in children. The relationship between sex (females) and voriconazole plasma concentration in adults was examined in two studies. We pooled the coefficients and standard errors from the children and adults, respectively. The pooled coefficients for this analysis were −0.16 (95% CI: −0.46 to 0.14) for children and −0.08 (95% CI: −0.44 to 0.28) for adults, but these results were not statistically significant.

#### 3.5.3 Weight

In the children cohort and the adult cohort, respectively, three studies reported both coefficients and standard errors from multiple linear regression analyses. When these coefficients and standard errors were pooled separately, the resulting coefficients were −0.01 (95% CI: −0.05 to 0.02) for children and −0.01 (95% CI: −0.03 to 0.01) for adult, both of which lacked statistical significance.

#### 3.5.4 Voriconazole dose

The voriconazole doses administered to adults and children varied, requiring separate pooling of the adult dose, the dose for children, and their respective plasma concentrations. The pooled coefficients for dose (mg) and dose (mg/kg) were 0.01 (95% CI: 0.00–0.02) and 0.23 (95% CI: −0.15 to 0.62), neither of which was statistically significant.

#### 3.5.5 Route of administration

This study investigated the correlation between plasma voriconazole concentration and intravenous administration in children, as well as the correlation with oral administration in adults. The pooled coefficients for these were −0.02 (95% CI: −0.58 to 0.80) and −0.72 (95% CI: −1.63 to 0.19), respectively, with no statistical significance observed in either case.

#### 3.5.6 Albumin

Only two studies confirmed the relationship between albumin and voriconazole plasma concentration in children. The pooled coefficient was −0.05 (95% CI: −0.09 to −0.01), indicating a statistically significant association.

#### 3.5.7 Liver function

Several indicators of liver function were considered, but the study focused on the correlations between TBil, ALT, AST, and voriconazole concentration in children. The pooled coefficients for these indicators were 0.17 (95% CI: −0.30 to 0.65), 0.00 (95% CI: −0.01 to 0.00), and 0.01 (95% CI: 0.00–0.01), respectively. However, none showed statistically significant correlations.

#### 3.5.8 Drug combinations

Three studies explored the correlation between PPIs and voriconazole plasma concentration in adults, but the pooled estimate did not show statistical significance. For specific PPIs, two studies focused on omeprazole in relation to voriconazole plasma concentration in adults, and another two on pantoprazole in relation to voriconazole plasma concentration in adults. The pooled coefficients were 1.37 (95% CI 0.82–1.92) for omeprazole and 1.11 (95% CI: 0.17–2.04) for pantoprazole. These results suggest that omeprazole and pantoprazole increase voriconazole plasma concentration by 1.37-fold and 1.11-fold, respectively.

Two studies on glucocorticoids were pooled to assess their association with plasma voriconazole concentration in children. The pooled coefficient was −0.51 (95% CI: −1.66 to 2.67). Three studies on glucocorticoids were pooled to assess their association with plasma voriconazole concentration in adults. The pooled coefficient was −1.33 (95% CI: −3.25 to 0.58). But the results all did not show statistical significance. Two studies focused on methylprednisolone in correlation to voriconazole plasma concentration in adults, and another two on dexamethasone in correlation to voriconazole plasma concentration in adults. The pooled coefficients were −1.75 (95% CI: −2.21 to −1.30) for methylprednisolone and −1.45 (95% CI: −2.07 to −0.83) for dexamethasone, indicating that the voriconazole plasma concentration decreased by 1.75-fold and 1.45-fold, respectively, with the concurrent use of these glucocorticoids in adults.

#### 3.5.9 Inflammation and genetic factor

Only one study established a correlation between C-reactive protein (CRP) and plasma voriconazole concentration; therefore, we did not pool the coefficient and standard error for this parameter. Among the 20 studies, 11 reported a correlation between the CYP2C19 genotype or phenotype and plasma voriconazole concentration. However, the coefficients and standard errors of these studies were not pooled.

### 3.6 Publication bias

We used the Egger test, the Begg test, and the trim-and-fill method to assess publication bias in the pooled estimate of the prevalence of patients reaching the therapeutic range of plasma voriconazole concentration. Begg’s test yielded a result of 0.127, and Egger’s test showed 0.045. Although the Egger test result did not exceed the threshold of 0.05, it was close to this limit. Consequently, the trim-and-fill method was applied to further the analysis. Using the linear method with two iterations, the software estimated that no studies were missing, suggesting an absence of publication bias. The detailed results of the trim-and-fill method are presented in in Supplementary Figure S2.

## 4 Discussion

The high pharmacokinetic variability of voriconazole and the benefits of maintaining its’ plasma concentration within the therapeutic range to improve efficacy and safety are well recognized. This study represents the first effort to pool data on the prevalence of patients who achieve this therapeutic range, underscoring the vital importance of TDM in voriconazole. Our analysis of the factors that influence plasma voriconazole concentration offers valuable evidence to healthcare decision-makers, helping to develop more effective voriconazole treatment strategies.

In the studies included in our analysis, the prevalence of patients reaching the therapeutic range of voriconazole plasma concentration varied widely, ranging from 33% to 84%. This variance can be partly attributed to the recommended therapeutic range of voriconazole plasma concentration varies in different regions. For example, guidelines in Japan, the United Kingdom, Australia, and the United States typically recommend a range of 1.0–5.5 mg/L for voriconazole (Ashbee et al., 2014; Hamada et al., 2013; Pappas et al., 2016; Chau et al., 2021), while the Canadian guideline suggests 1.5–5.0 mg/L (Laverdiere et al., 2014), and in China, it is defined as 0.5–5.5 mg/L (Chen et al., 2018). Our study included studies that adopted nine different therapeutic ranges for plasma voriconazole concentration. Although the therapeutic range, sex, and the analytical method for voriconazole measurement were identified as significant factors affecting heterogeneity through meta-regression, other contributors could not be completely excluded. These may include variations in treatment duration, types of treatment settings, and the purpose of voriconazole use (prophylaxis or treatment), all of which could influence achieving the effective and safe therapeutic concentration. Additionally, disparities in study design and patient populations might also affect the prevalence of patients reaching the effective and safe therapeutic range of voriconazole concentrations.

The pooled prevalence was higher in studies with dose adjustments in accordance with TDM and guidelines than in studies without dose adjustments. The results suggested that dose adjustment according to TDM and guidelines is helpful to improve the prevalence of patients reaching concentrations within the effective and safe range. Since most of the studies were retrospective, many of them only mentioned the CYP2C19 genotype testing, but did not mention whether the dose was adjusted according to the genotype. This prevented us from determining the effect of dose adjustment based on CYP2C19 genotype on patients achieving therapeutic range. After subgroup analysis according to the study population, the pooled prevalence of adult patients reaching the therapeutic range is higher than that of children. In adults, the physiological maturity and stability facilitate achieving the effective and safe therapeutic range of voriconazole plasma concentration. In contrast, children exhibit a lower prevalence of reaching the target concentration due to the incomplete development of their metabolic enzyme systems, variations in drug distribution and absorption, and greater individual physiological variability.

Numerous factors influence plasma voriconazole concentration, as highlighted in our study, which included 21 studies that performed multiple linear regression analyses to explore these relationships. Factors associated with plasma voriconazole concentration identified in the literature included primarily demographic characteristics (age, sex, weight, ethnicity, etc.), genetic factors (such as CYP2C19 gene polymorphism), drug interactions (with PPIs, glucocorticoids, etc.), administration methods (oral, intravenous), and dosage. However, not all of these factors were found to have a significant correlation with plasma voriconazole concentration. Although our study did not pool coefficients and standard errors between the CYP2C19 genotype or phenotype and voriconazole plasma concentration, the impact of CYP2C19 on voriconazole plasma concentration is well established. A clinical study has shown that the plasma concentration of voriconazole is influenced by CYP2C19 genotype in both children and adults (Hu et al., 2023). CYP2C19 is crucial in voriconazole metabolism and exhibits a significant genetic polymorphism (Dean, 2012). Poor metabolizers show an approximately four-fold increase in voriconazole exposure compared to homozygous normal metabolizers (Dean, 2012). Heterozygous normal metabolizers experience, on average, a two-fold increase in exposure compared to their homozygous normal counterparts (Dean, 2012). Single nucleotide polymorphisms (SNPs) in the CYP2C19 gene account for approximately 50%–55% of variability in voriconazole metabolism (Murayama et al., 2007). Both the Clinical Pharmacogenetics Implementation Consortium (CPIC) and the Dutch Pharmacogenetics Working Group (DWPG) recommend adjusting the voriconazole dosing regimen based on the CYP2C19 genotype (Moriyama et al., 2017; Swen et al., 2011). Therefore, it is recommended that both adults and children should adjust the voriconazole dosing regimen based on CYP2C19 genotype.

We observed a weak correlation between age and voriconazole plasma concentration in children, but not in adults. This difference may be attributed to differences in drug metabolism and physiological characteristics between the two groups. Moreover, since children’s physiological and metabolic systems are not fully developed, their response to medication may change as they age. A systematic review showed that the dosing of voriconazole in pediatric patients was age-dependent, with younger children requiring higher doses to reach target concentration compared to older children (Hu et al., 2024).

Several studies have highlighted the impact of inflammation on voriconazole metabolism (Liang et al., 2022; Bolcato et al., 2021). Inflammatory cytokines, such as IL-6 and IL-8, can modulate drug-metabolizing enzymes and transporters (DMETs) (Stanke-Labesque et al., 2020), affecting the expression of CYP2C19 and CYP34A, influencing voriconazole plasma concentration (Vreugdenhil et al., 2018; Gautier-Veyret et al., 2017; Klein et al., 2015; Li et al., 2014). CRP, an inflammation indicator, was shown to influence the plasma concentration of voriconazole by impacting CYP2C19 activity (Van Wanrooy et al., 2014; Ruiz et al., 2019; Simon et al., 2021). However, our analysis was limited by the small number of studies addressing CPR, IL-6, and IL-8. Consequently, we could not pool the coefficients from multiple linear regressions for a random-effects model. A systematic review indicated that the level of inflammation (CRP levels) can significantly affect voriconazole plasma concentration in adults (Li et al., 2022). However, a clinical study identified variations in the correlation between CRP levels and voriconazole plasma concentration among pediatric patients across different age groups (Luo et al., 2021). The impact of inflammation (CRP levels) on voriconazole metabolism in children should need further analysis in relation to age groups, which requires a further exploration with a larger sample size.

In our analysis, we found a weak correlation between albumin and voriconazole plasma concentrations in children. Regarding the correlation between albumin and plasma concentration of voriconazole in adults, this article could not conduct research, but some studies showed that hypoproteinemia affected the plasma concentration of voriconazole in adults (Li et al., 2023; Chantharit et al., 2020; Khan-Asa et al., 2020). The inflammatory cytokine IL-6 increased CRP synthesis and decreased albumin synthesis in the liver (Bologa et al., 1998), likely leading to higher voriconazole plasma concentrations due to elevated CRP levels rather than reduced albumin.

Voriconazole is predominantly metabolized in the liver through oxidative processes mediated by CYP450 enzymes, including CYP2C19, CYP3A4, and CYP2C9 (Ashbee et al., 2014). Liver injury may cause metabolic abnormalities and increased exposure, affecting plasma voriconazole concentration. Hepatocellular injury is often indicated by elevated levels of ALT and AST (Kwo et al., 2017). Due to the exclusion of patients with abnormal liver function in some included studies and the limited number of studies that conducted multiple linear regression to assess correlations between AST, ALT, Child-Pugh class, and plasma voriconazole concentration, our study did not observe a definitive correlation between these liver function indicators and plasma voriconazole concentration in the children and adult population. However, we recommend dose reduction or avoidance of voriconazole in patients with impaired liver function (Hamada et al., 2013; Limper et al., 2011). The Japanese guideline (Hamada et al., 2013) recommends maintaining the loading dose but halving the maintenance dose for patients with severe liver disease classified as Child-Pugh classes A and B, while voriconazole is not recommended for those in class C. Similarly, United Kingdom, Canada, the United States, and Australia guidelines generally do not recommend antifungal therapy for patients with severe liver insufficiency (Ashbee et al., 2014; Laverdiere et al., 2014; Chau et al., 2021; Pappas et al., 2016). The Chinese guideline (Chen et al., 2018) advises against using voriconazole as an initial therapy for patients with severe liver disease, recommending close monitoring of plasma concentration and liver function if its use is deemed necessary.

The correlation between plasma voriconazole concentration and PPIs in adults were not found in our study, however, we found that voriconazole plasma concentration was positively correlated with omeprazole and pantoprazole in adults. Additionally, we also did not observe the relationship between plasma voriconazole concentration and glucocorticoids in children and adults, however, a negatively correlated between methylprednisolone, dexamethasone and voriconazole plasma concentration in adults was found in our study. Voriconazole inhibits the activity of CYP2C19, CYP3A4, and CYP2C9, resulting in several clinically significant drug interactions (Ashbee et al., 2014). PPIs(Klotz et al., 2004; Li et al., 2004) and glucocorticoids (Czock et al., 2005; Chen et al., 2003) are known to affect CYP2C19 activity. However, neither PPIs nor glucocorticoids seem to affect the pharmacokinetics of voriconazole (Shi et al., 2019). Some studies indicated that glucocorticoids and PPIs can impact plasma voriconazole concentration (Jia et al., 2021; Li et al., 2004). Rabeprazole and lansoprazole are less potent CYP450 inhibitors compared to omeprazole and pantoprazole (Li et al., 2004), and another study found that voriconazole plasma concentration decreased significantly with dexamethasone or methylprednisolone, but less so with prednisone or prednisolone (Jia et al., 2021). Our analysis did not find a correlation between the PPIs as a group, glucocorticoids as a group and plasma voriconazole concentration, possibly due to the inclusion of different types of PPIs and glucocorticoids in the reviewed studies. In summary, the effects of PPIs and glucocorticoids on voriconazole pharmacokinetics are disputed. Indeed, substantial studies showed that the type of PPI and dose (or glucocorticoid) significantly impact on voriconazole exposure (Qi et al., 2017; Jia et al., 2021). Drug interactions may occur in both adults and children; thus, attention should be paid to the combined use of glucocorticoids or PPIs, including consideration of the type and dose of these medications.

Although our study provides a comprehensive quantitative summary of the prevalence of patients who achieve the therapeutic range of plasma voriconazole concentration and associated factors, it is important to recognize several limitations. First, the included studies covered a global scale, including various medical practices, economic conditions, geographic locations, and cultural backgrounds. These factors could influence the reported prevalence of patients who reached the therapeutic voriconazole concentration. Second, the studies exhibited high heterogeneity, possibly due to variations in sample size, study populations, sex distribution, indications for voriconazole use, dose adjustments, and concentration measurement methodologies. Third, the restriction to studies published in English might have led to an underestimation or overestimation of prevalence and limited the pooling of several factors. Some factors in our study were only analyzed in two studies, raising concerns about possible bias in these results. Therefore, further research is warranted to better understand the factors associated with voriconazole plasma concentration.

## 5 Conclusion

The analysis revealed that only approximately half of the patients reached the plasma voriconazole concentration therapeutic range without dose adjustments and the pooled prevalence of adult patients reaching the therapeutic range is higher than that of children. The meta-analysis identified factors associated with voriconazole plasma concentration in children, including age, albumin levels. Meanwhile, it also identified the factors related to plasma voriconazole concentration in adults, including the use of omeprazole, pantoprazole, dexamethasone and methylprednisolone.

## Data Availability

The original contributions presented in the study are included in the article/Supplementary Material, further inquiries can be directed to the corresponding author.
