# Supplementary material for: Associated factors with voriconazole plasma concentration: a systematic review and meta-analysis
Source: Front Pharmacol. 2024 Aug 23;15:1368274. doi: 10.3389/fphar.2024.1368274 (PMC11377273; doi:10.3389/fphar.2024.1368274)
Supplement: Supplementary file 1 [file DataSheet1.docx]

Supplemental Table S1 – Search strategy

| Database | String |
| --- | --- |
| PubMed | #1 "Drug Monitoring"[Mesh]  #2 (Monitoring, Drug[Title/Abstract]) OR (Therapeutic Drug Monitoring[Title/Abstract]) OR (Drug Monitoring, Therapeutic[Title/Abstract]) OR (Monitoring, Therapeutic Drug[Title/Abstract])  #3 plasma concentrations[Title/Abstract]  #4 #1 OR #2 OR #3  #5 "Voriconazole"[Mesh]  #6 ("Voriconazole"[Mesh]) OR ((UK 109,496[Title/Abstract]) OR (UK-109,496[Title/Abstract]) OR (UK-109496[Title/Abstract]) OR (UK109,496[Title/Abstract]) OR (UK109496[Title/Abstract]) OR (UK 109496[Title/Abstract]) OR (Vfend[Title/Abstract]))  # 7 #5 OR #6  #8 #4 AND #7 |
| Embase | 1 exp drug monitoring/ 61157  2 plasma concentrations.mp. [mp=title, abstract, heading word, drug trade name, original title, device manufacturer, drug manufacturer, device trade name, keyword heading word, floating subheading word, candidate term word] 71059  3 (Monitoring, Drug or Therapeutic Drug Monitoring or Drug Monitoring, Therapeutic or Monitoring, Therapeutic Drug).mp. [mp=title, abstract, heading word, drug trade name, original title, device manufacturer, drug manufacturer, device trade name, keyword heading word, floating subheading word, candidate term word] 18845  4 1 or 2 or 3 134583  5 exp voriconazole/ 26282  6 (UK 109,496 or UK-109,496 or UK-109496 or UK109,496 or UK10949 or UK 109496 or Vfend).mp. [mp=title, abstract, heading word, drug trade name, original title, device manufacturer, drug manufacturer, device trade name, keyword heading word, floating subheading word, candidate term word] 784  7 5 or 6  8 4 and 7 |
| Web of science | 1: Drug Monitoring (Topic) OR Monitoring, Drug (All Fields) OR Therapeutic Drug Monitoring (All Fields) OR Drug Monitoring, Therapeutic (All Fields) OR Monitoring, Therapeutic Drug (All Fields) OR plasma concentrations (All Fields)  2: Voriconazole (Topic) OR UK 109,496 (All Fields) OR UK-109,496 (All Fields) OR UK109,496 (All Fields) OR UK-109496 (All Fields) OR UK109496 (All Fields) OR UK 109496 (All Fields) OR Vfend (All Fields)  3: #1 AND #2  4: #1 AND #2 |
| Cochrane Library | #1MeSH descriptor: [Drug Monitoring] explode all trees  #2 (Monitoring, Drug):ti,ab,kw OR (Therapeutic Drug Monitoring):ti,ab,kw OR  (Drug Monitoring, Therapeutic):ti,ab,kw OR (Monitoring, Therapeutic Drug):ti,ab,kw  #3 plasma concentrations  #4 #1 OR #2 OR #3  #5 MeSH descriptor: [Voriconazole] explode all trees  #6 (Vfend):ti,ab,kw OR (UK109496):ti,ab,kw OR (UK109,496):ti,ab,kw OR  (UK109,496):ti,ab,kw OR (UK109,496):ti,ab,kw OR (UK-109496):ti,ab,kw OR  (UK 109496):ti,ab,kw OR (UK 109,496):ti,ab,kw  #7 #5 OR #6  #8 #4 AND #7 |

Table S2 Details of dose adjustments.

| Study | Dose adjustments | Guideline for dose adjustment |
| --- | --- | --- |
| Aiuchi (2022) | No | No |
| Bartelink (2013) | Yes | TDM |
| Benedict (2023) | Yes | NA |
| Blanco-Dorado (2019) | No | No |
| Boast (2016) | Yes | TDM |
| Cabral-Galeano (2015) | Yes | TDM |
| Chaudhri (2020) | Yes | Australian national guidelines |
| Chen (2022) | No | No |
| Chen (2022) | Yes | NA |
| Chen (2022) | Yes | Chinese guideline |
| Chen (2022) | No | No |
| Cheng (2020) | Yes | NA |
| Choi (2013) | Yes | TDM |
| Chuwongwattana (2016) | NA | / |
| Dorado (2020) | No | No |
| Duehlmeyer (2021) | Yes | TDM |
| Ebrahimpour (2017) | No | No |
| Hoenigl(2013) | Yes | NA |
| Hu (2018) | Yes | TDM |
| Hu (2023) | Yes | TDM |
| Huang (2023) | Yes | NA |
| Huang (2020) | Yes | NA |
| Jia (2021) | Yes | Chinese guideline |
| Kang (2015) | Yes | TDM |
| Kim (2014) | Yes | NA |
| Lempers (2019) | Yes | TDM |
| Li (2020) | No | No |
| Li (2023) | Yes | NA |
| Liu (2017) | No | No |
| Miao (2019) | Yes | NA |
| Myrianthefs (2010) | No | No |
| Pieper (2012) | NA | / |
| Ronda (2023) | Yes | NA |
| Ruiz (2019) | No | No |
| Saini (2014) | No | No |
| Shao (2017) | Yes | NA |
| Shen (2022) | Yes | TDM |
| Soler-Palacin (2012) | Yes | TDM |
| Takahashi (2020) | No | No |
| Tian (2021) | Yes | Chinese guideline |
| Troke (2011) | Yes | TDM |
| Valle-T-Figueras (2021) | Yes | TDM |
| Wei (2019) | NA | / |
| Yan (2018) | No | No |
| Yang (2023) | No | No |
| Ye (2022) | Yes | TDM |
| Yi (2017) | Yes | TDM |
| Zeng (2020) | Yes | NA |
| Zhang (2023) | No | No |
| Zhao (2021) | Yes | TDM |
| Zhao (2021) | Yes | NA |
| Zhou (2019) | No | No |

Table S3 Quality assessment of Cross-sectional study

| S.no | (Author, year of pub.) | the source of data | eligible criteria for study subjects | time period for included population | whether or not subjects were consecutive | evaluators were masked to other aspects of the status of the participants | any assessments for quality assurance | explanation for excluding any patients from analysis | measurements taken for controlling confound factors | description for the handing of missing data | summary for patient response rate and completeness of data collection | clarification of following-up results | Total Score |
| --- | --- | --- | --- | --- | --- | --- | --- | --- | --- | --- | --- | --- | --- |
| 1 | Aiuchi 2022 | 1 | 1 | 1 | 1 | 0 | 1 | 1 | 1 | 0 | 1 | 0 | 8 |
| 2 | Allergra2018 | 1 | 1 | 1 | 1 | 0 | 1 | 1 | 1 | 0 | 1 | 0 | 8 |
| 3 | Bartelink 2013 | 1 | 1 | 1 | 1 | 0 | 1 | 0 | 0 | 0 | 1 | 0 | 6 |
| 4 | Benedict 2023 | 1 | 1 | 1 | 1 | 0 | 1 | 0 | 0 | 0 | 1 | 0 | 6 |
| 5 | Blanco-Dorado 2019 | 1 | 1 | 1 | 1 | 0 | 1 | 0 | 0 | 0 | 1 | 0 | 6 |
| 6 | Boast 2016 | 1 | 1 | 1 | 1 | 0 | 1 | 0 | 0 | 0 | 1 | 0 | 6 |
| 7 | Cabral-Galeano 2015 | 1 | 1 | 1 | 1 | 0 | 1 | 0 | 0 | 0 | 1 | 0 | 6 |
| 8 | Chaudhri 2020 | 1 | 1 | 1 | 1 | 0 | 1 | 0 | 0 | 0 | 1 | 0 | 6 |
| 9 | Chen 2020 | 1 | 1 | 1 | 1 | 0 | 1 | 0 | 1 | 0 | 1 | 0 | 7 |
| 10 | Chen 2022 | 1 | 1 | 1 | 1 | 0 | 1 | 0 | 1 | 0 | 1 | 0 | 7 |
| 11 | Chen 2022 | 1 | 1 | 1 | 1 | 0 | 1 | 0 | 1 | 0 | 1 | 0 | 7 |
| 12 | Chen 2022 | 1 | 1 | 1 | 1 | 0 | 1 | 0 | 1 | 0 | 1 | 0 | 7 |
| 13 | Cheng 2020 | 1 | 1 | 1 | 1 | 0 | 1 | 0 | 1 | 0 | 1 | 0 | 7 |
| 14 | Choi 2013 | 1 | 1 | 1 | 1 | 0 | 1 | 0 | 0 | 0 | 1 | 0 | 6 |
| 15 | Chuwongwattana 2016 | 1 | 1 | 1 | 1 | 0 | 1 | 0 | 0 | 0 | 1 | 0 | 6 |
| 16 | Cojutti 2016 | 1 | 1 | 1 | 1 | 0 | 1 | 0 | 1 | 0 | 1 | 0 | 7 |
| 17 | Dolton 2012 | 1 | 1 | 1 | 1 | 0 | 1 | 0 | 1 | 0 | 1 | 0 | 7 |
| 18 | Dorado 2020 | 1 | 1 | 1 | 1 | 0 | 1 | 0 | 1 | 0 | 1 | 0 | 7 |
| 19 | Dote 2016 | 1 | 1 | 1 | 1 | 0 | 1 | 1 | 1 | 0 | 1 | 0 | 8 |
| 20 | Duehlmeyer 2021 | 1 | 1 | 1 | 1 | 0 | 1 | 0 | 0 | 0 | 1 | 0 | 6 |
| 21 | Ebrahimpour 2017 | 1 | 1 | 1 | 1 | 0 | 1 | 1 | 0 | 0 | 1 | 0 | 7 |
| 22 | Fan 2020 | 1 | 1 | 1 | 1 | 0 | 1 | 1 | 0 | 0 | 1 | 0 | 7 |
| 23 | Hashemizadeh 2017 | 1 | 1 | 1 | 1 | 0 | 1 | 1 | 1 | 0 | 1 | 0 | 8 |
| 24 | Hoenigl 2013 | 1 | 1 | 1 | 1 | 0 | 1 | 0 | 1 | 0 | 1 | 0 | 7 |
| 25 | Hu 2018 | 1 | 1 | 1 | 1 | 0 | 1 | 0 | 1 | 0 | 1 | 0 | 7 |
| 26 | Hu 2023 | 1 | 1 | 1 | 1 | 0 | 1 | 0 | 1 | 0 | 1 | 0 | 7 |
| 27 | Huang 2023 | 1 | 1 | 1 | 1 | 0 | 1 | 0 | 0 | 0 | 1 | 0 | 6 |
| 28 | Huang 2020 | 1 | 1 | 1 | 1 | 0 | 1 | 0 | 0 | 0 | 1 | 0 | 6 |
| 29 | Jia 2021 | 1 | 1 | 1 | 1 | 0 | 1 | 0 | 0 | 0 | 1 | 0 | 6 |
| 30 | Kang 2015 | 1 | 1 | 1 | 1 | 0 | 1 | 0 | 0 | 0 | 1 | 0 | 6 |
| 31 | Kim 2014 | 1 | 1 | 1 | 1 | 0 | 1 | 1 | 0 | 0 | 1 | 0 | 7 |
| 32 | Lempers 2019 | 1 | 1 | 1 | 1 | 0 | 1 | 0 | 0 | 0 | 1 | 0 | 6 |
| 33 | Li 2020 | 1 | 1 | 1 | 1 | 0 | 1 | 0 | 1 | 0 | 1 | 0 | 7 |
| 34 | Li 2023 | 1 | 1 | 1 | 1 | 0 | 1 | 0 | 1 | 0 | 1 | 0 | 7 |
| 35 | Liu 2017 | 1 | 1 | 1 | 1 | 0 | 1 | 0 | 0 | 0 | 1 | 0 | 6 |
| 36 | Mafuru 2021 | 1 | 1 | 1 | 1 | 0 | 1 | 0 | 1 | 0 | 1 | 0 | 7 |
| 37 | Miao 2019 | 1 | 1 | 1 | 1 | 0 | 1 | 0 | 0 | 0 | 1 | 0 | 6 |
| 38 | Myrianthefs 2010 | 1 | 1 | 1 | 1 | 0 | 1 | 0 | 0 | 0 | 1 | 0 | 6 |
| 39 | Pieper 2012 | 1 | 1 | 1 | 1 | 0 | 1 | 0 | 0 | 0 | 1 | 0 | 6 |
| 40 | Ronda 2023 | 1 | 1 | 1 | 1 | 0 | 1 | 0 | 1 | 0 | 1 | 0 | 7 |
| 41 | Ruiz 2019 | 1 | 1 | 1 | 1 | 0 | 1 | 0 | 1 | 0 | 1 | 0 | 7 |
| 42 | Saini 2014 | 1 | 1 | 1 | 1 | 0 | 1 | 0 | 0 | 0 | 1 | 0 | 6 |
| 43 | Shao 2017 | 1 | 1 | 1 | 1 | 0 | 1 | 0 | 1 | 0 | 1 | 0 | 7 |
| 44 | Shen 2022 | 1 | 1 | 1 | 1 | 0 | 1 | 0 | 0 | 0 | 1 | 0 | 6 |
| 45 | Soler-Palacin 2012 | 1 | 1 | 1 | 1 | 0 | 1 | 0 | 0 | 0 | 1 | 0 | 6 |
| 46 | Takahashi 2020 | 1 | 1 | 0 | 0 | 0 | 1 | 0 | 1 | 0 | 1 | 0 | 5 |
| 47 | Tian 2021 | 1 | 1 | 1 | 1 | 0 | 1 | 0 | 1 | 0 | 1 | 0 | 7 |
| 48 | Troke 2011 | 1 | 1 | 1 | 0 | 0 | 1 | 1 | 0 | 0 | 1 | 0 | 6 |
| 49 | Valle-T-Figueras 2021 | 1 | 1 | 1 | 1 | 0 | 1 | 0 | 0 | 0 | 1 | 0 | 5 |
| 50 | Wei 2019 | 1 | 1 | 1 | 1 | 0 | 1 | 0 | 0 | 0 | 1 | 0 | 6 |
| 51 | Yan 2018 | 1 | 1 | 1 | 1 | 0 | 1 | 0 | 1 | 0 | 1 | 0 | 7 |
| 52 | Yang 2023 | 1 | 1 | 1 | 1 | 0 | 1 | 0 | 1 | 0 | 1 | 0 | 7 |
| 53 | Ye 2022 | 1 | 1 | 1 | 1 | 0 | 1 | 0 | 1 | 0 | 1 | 0 | 7 |
| 54 | Yi 2017 | 1 | 1 | 1 | 1 | 0 | 1 | 0 | 1 | 0 | 1 | 0 | 7 |
| 55 | Zeng 2020 | 1 | 1 | 1 | 1 | 0 | 1 | 0 | 1 | 0 | 1 | 0 | 7 |
| 56 | Zhang 2023 | 1 | 1 | 1 | 1 | 0 | 1 | 0 | 1 | 0 | 1 | 0 | 7 |
| 57 | Zhao 2021 | 1 | 1 | 1 | 1 | 0 | 1 | 0 | 1 | 0 | 1 | 0 | 7 |
| 58 | Zhao 2021 | 1 | 1 | 1 | 1 | 0 | 1 | 0 | 1 | 0 | 1 | 0 | 7 |
| 59 | Zhao 2021 | 1 | 1 | 1 | 1 | 0 | 1 | 0 | 1 | 0 | 1 | 0 | 7 |
| 60 | Zhou 2019 | 1 | 1 | 1 | 1 | 0 | 1 | 0 | 0 | 0 | 1 | 0 | 6 |


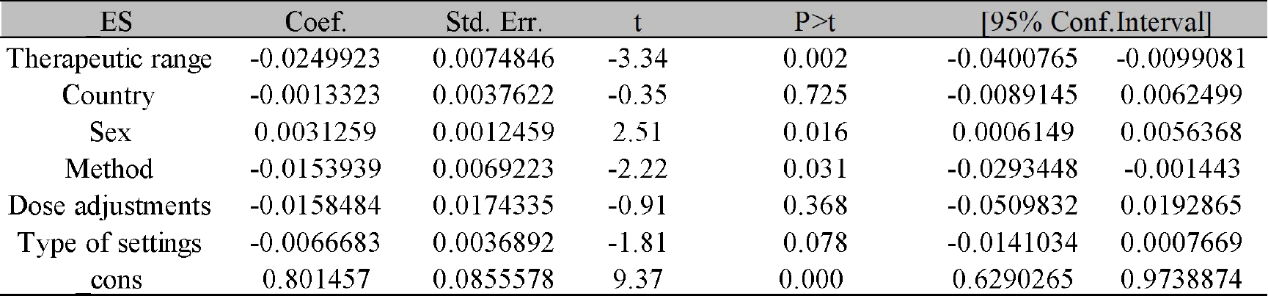


Figure S1 Meta regression.


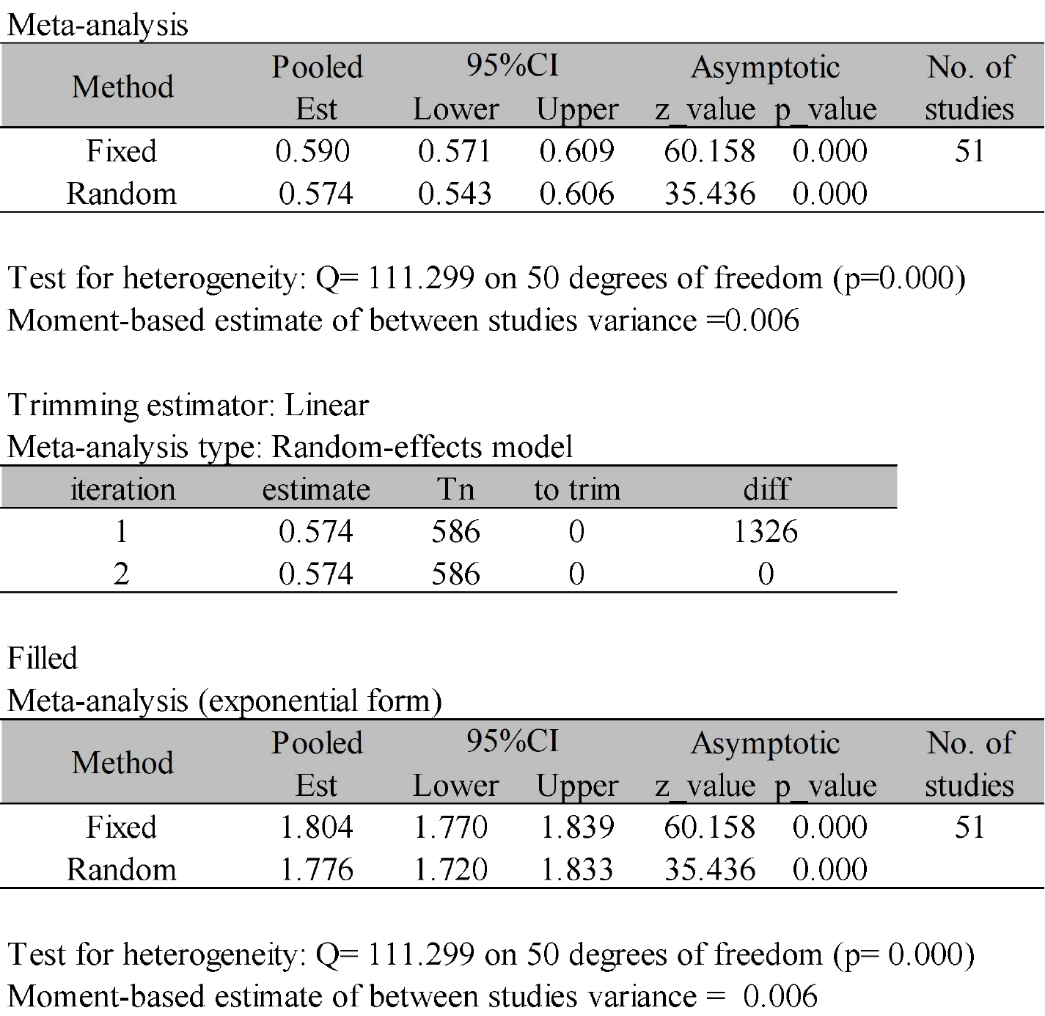


Figure S2 Trim-and-fill method.
